# Influence of Different Powder Conditioning Strategies on Metal Binder Jetting with Ti-6Al-4V

**DOI:** 10.3390/ma17030750

**Published:** 2024-02-04

**Authors:** Kevin Janzen, Kim Julia Kallies, Lennart Waalkes, Philipp Imgrund, Claus Emmelmann

**Affiliations:** 1 Fraunhofer Research Institution for Additive Production Technologies IAPT, Am Schleusengraben 14, 21029 Hamburg, Germany; lennart.waalkes@iapt.fraunhofer.de (L.W.); philipp.imgrund@iapt.fraunhofer.de (P.I.); 2 Faculty 2: Computer Science and Engineering, Frankfurt University of Applied Sciences, Nibelungenpl. 1, 60318 Frankfurt am Main, Germany; kallies@stud.fra-uas.de; 3 Institute of Laser and System Technologies (iLAS), Hamburg University of Technology TUHH, 21073 Hamburg, Germany; c.emmelmann@tuhh.de

**Keywords:** metal binder jetting, additive manufacturing, powder conditioning, titanium alloys

## Abstract

Metal binder jetting shows great potential for medical technology. This potential can be exploited by integrating binder jetting into existing process routes known from metal injection molding. The biggest challenge here is the flowability and packing behavior of the powders used, due to their low size distributions. This paper investigates different powder-drying strategies to improve flowability using a statistical experimental design. Because of its relevance for medical applications, spherical Ti-6Al-4V powder with a size distribution under 25 µm is dried under various parameters using vacuum and gas purging. The investigated parameters, time and temperature, are selected in a central-composite-circumscribed test plan with eleven tests and three center points. The target parameters—water content, flowability and impurity levels (oxygen, nitrogen)—of the powder are analyzed. For validation, practical test trials are carried out on an industrial binder jetting system with unconditioned powder and conditioning with optimized parameters, comparing the manufactured parts and the powder bed. An optimized drying cycle with a duration of 6 h at 200 °C was determined for the investigated powder. Significant improvements in the dimensional accuracy (from ±1.5 to 0.3%) of the components and the visual impression of the powder bed are demonstrated.

## 1. Introduction

Additive manufacturing (AM) offers new possibilities for patient-specific and personalized products in medical technology. There are already a number of applications for the widely used laser and electron-beam powder bed fusion (L/E-PBF), e.g., in the additive manufacturing of hip or knee implants [1,2,3]. Nevertheless, apart from these few very obvious medical technology use cases, AM technologies have not yet become widespread. Prostheses and implants with smaller accessible markets, like finger implants, are currently not economically feasible. The biggest hurdles continue to be the high investment that is required and, above all, the production and material costs [4]. With the market launch of the first metal binder jetting (MBJ) systems for industrial use, potential cost reductions were expected due to the elimination of expensive laser technology and the use of more cost-effective materials from metal injection molding (MIM), as well as seamless integration into similar process chains [5]. In contrast to L/E-PBF, in the MBJ process, metal particles are not fused by means of an energy input but are simply bonded using a liquid binder. This is followed by a debinding and sintering of the so-called green part, whereby the binder is removed and the metal particles enter the metallic bonds via diffusion processes and form a nearly dense component [6,7,8]. Despite the fact that the reduction in component costs seems possible and that medical technology in particular offers many promising applications for MBJ [9,10], this breakthrough has not yet been achieved [4]. Not only are the necessary investment costs still in comparable regions to those of the established L/E-PBF system manufacturers [11], there is also a lack of medically certified process routes for biomaterials like titanium, as well as suitable powder conditioning strategies to directly use MIM powders to integrate binder jetting into the corresponding process routes.

The most relevant material for biomedical applications and prostheses at present is titanium and its alloys because they have characteristics like biocompatibility and non-toxicity, and favorable mechanical properties [12,13]. MBJ, compared to fusion-based AM technologies like L-PBF or E-PBF, shows clear advantages in the manufacturing of titanium alloys, specifically for creating personalized biomedical devices. Especially intricate parts can be produced without the need for support structures. According to the literature, compared to L- and E-PBF, MBJ avoids thermal stresses, prevents shape distortion and cracking, and does not induce undesired microstructural features or material loss, ensuring high material recycling efficiency and cost-effectiveness, particularly for costly materials [6,14]. Despite these advantages, there is a lack of comprehensive literature available on MBJ regarding titanium and its alloys and the processing of fine MIM powders.

İyibilgin et al. demonstrated a density of around 92% through sintering CP-Ti parts at 1200 °C in an argon environment, highlighting the challenges presented by the lower density in the manufactured parts, resulting in reduced mechanical strength compared to conventional powder metallurgy methods [15]. Basalah et al. explored the effects of layer thickness (62–175 µm) and sintering temperature (800–1400 °C) on CP-Ti microstructure and densification. They managed to create porous samples (17–44%) that are suitable as a bone substitute, offering compressive strengths ranging from 27 to 509 MPa [16,17]. Sheydaeian et al. investigated the MBJ of CP-Ti, emphasizing the complexities associated with particle segregation during powder deposition and anisotropic shrinkage during sintering [18]. Wheat et al.’s findings confirmed these challenges, using computer tomography to visualize how particle size distribution affected the density and microstructure of green and sintered CP-Ti samples [19]. Polozov et al. attempted to fabricate Ti-22Al-25Nb alloy via the MBJ of mixed elemental powders, high-temperature reactive sintering (800–1100 °C), and subsequent annealing at 1400 °C. The resulting microstructure showcased a B2/β-phase with acicular Ti2AlNb precipitates [20]. Miyanaji et al. proposed a physics-based model predicting the optimal saturation level for the binder jet printing of Ti-6Al-4V alloy, although its application in functional part fabrication remains unexplored beyond experimental verification [21]. Stevens et al. examined the Ti-6Al-4V alloy MBJ, revealing a varying sintered density across different structural areas due to powder–binder interactions during printing [22]. Simchi et al. explored the challenges in fabricating biomedical-grade titanium alloys using MBJ. They successfully demonstrated strategies to minimize pore formation during printing, achieving high-density Ti-6Al-4V parts comparable to commercial alloys, and emphasizing the potential of MBJ in custom biomaterial development. The carbon content (less than 0.02 wt.%) and nitrogen content (0.01 wt.%) aligned with the standard range for MIM. Nonetheless, sintering led to a moderate increase in oxygen content (by 30–50%) beyond the standard level [23]. Barthel et al. explored the influence of powder condition and spreading parameters on green and sintered density in MBJ for stainless steel 316L. Powder drying enhanced the statistical impact of spreading parameters, improving green density without affecting sintered density; layer thickness and roller diameter were the most impactful factors, with combinations leading to a high green density also yielding a high sintered density [24]. Zissel et al. assessed the reusability of 17–4PH stainless steel powder in MBJ, highlighting how changes in the powder properties and surface conditions impact the process’ reproducibility and the properties of the final part. They investigated the degradation of reused powder through multiple cycles involving various process steps, like drying, sieving, printing, and curing, comparing its influence on print quality and green densities to that of virgin powder. Additionally, their research examined the impact of inert and reducing atmospheres on the powder’s reusability during curing in the MBJ process [25].

One hurdle in the development of suitable sintering strategies for titanium alloys lies in the powder materials used for MBJ. Currently, because of their better flowability, powders with particle size distributions (PSD) of up to approx. 45 µm are generally used, which is above the typical PSD for MIM powders [7,26]. In addition to the use of other binder systems, this is the main reason why the integration of MBJ into MIM process routes causes difficulties. Due to the higher PSD that is required, users are forced to buy specific powder materials for MBJ despite the existing MIM process chain. Furthermore, the higher PSD reduces the sintering kinetics, which tends to lead to poorer material properties (especially residual porosities and coarser grain size), as well as longer sintering times [27]. In addition, MBJ components may not be able to be sintered together with MIM parts, which would have a negative effect on machine utilization. Miyanaji et al. investigated the potential of using fine copper powders in MBJ, examining their impact on green and sintered part properties. They explored how varying powder recoating settings and sintering parameters affect the density, shrinkage, microstructure, and mechanical characteristics of sintered specimens compared to copper specimens made from coarse powder materials, demonstrating that fine copper powder yields superior part properties (UTS of 179.4 MPa and elongation of 42.2%) compared to bimodal powder parts [28]. Bai et al. explored the use of bimodal powder mixtures in copper MBJ to improve part density and shrinkage control. Comparing mono-sized fine powders to bimodal mixtures, they found that the latter enhanced packing density (8.2%) and flowability (10.5%), increased sintered density (4.0%), and reduced sintering shrinkage (6.4%) [29]. Ziaee et al. investigated two methods of preparing fine 316 stainless steel powder for binder jetting, exploring their impact on sintered density and dimensions relative to direct printing into <22 µm powder. They revealed that while the sintered density and shrinkage of agglomerate materials vary with the density of the spread powder bed, incorporating nylon 12 powder as a space-holder allows for controlled porosity, enabling the creation of binder jetting systems with spatially controlled porosity while maintaining compatible shrinkage levels [30]. Du et al. explored the impact of particle size distribution on various densities in MBJ, also indicating that multimodal mixtures could achieve higher packing densities compared to individual powders. Analytical models revealed that an optimal mixing fraction leads to maximum mixture packing density, and the experimental findings aligned with these predictions, showcasing improved densities for powders with multimodal particle size distributions in most cases [31].

Patent EP3231536 A1 describes how to favorably influence the microstructure and mechanical properties of sintered titanium alloys by using powders with a size distribution of less than 25 µm and sintering at 1100 °C [32]. The authors did not find any studies on the use of MIM-typical powder size distributions below 25 µm for MBJ with Ti-6Al-4V at present. The following research article will therefore investigate possibilities for effective powder conditioning for the use of these powders in order to enable better integration into MIM process routes and create advantages for the subsequent sintering process.

## 2. Materials and Method

This chapter describes how the experiments were carried out. In addition to the actual experiments, the measuring equipment that was used is also explained in more detail and the necessary sampling is described. The room humidity and ambient temperature were documented several times during the entire test using hygrometers. Measurements were taken before the start and after the end of a test, as well as during sampling. The handling of the powder always took place under nitrogen in a glovebox. An oxygen measuring device was used to check that a maximum of 5% oxygen was present in the atmosphere.

### 2.1. Materials

In order to investigate powder conditioning with small particle size with a high relevance to medical technology, a plasma-atomized spherical Ti-6Al-4V powder from Tekna Advanced Materials, Inc., was selected, which is used industrially for the metal powder injection molding of medical components. The most important characteristic properties of the powder are shown in Table 1. Only powder from a single batch was used for the experiments.

### 2.2. Characterization

The greatest challenge when processing fine powders is the flowability and the associated negative influences on the quality of the powder bed. In addition, fine powders generally offer a larger surface area for passive oxidation. By investigating and optimizing the conditioning and drying strategy, the aim is to improve the flowability by reducing the water content of the powder under investigation. Therefore, a characterization of the water content and the flowability itself is carried out. The absorption of oxygen and nitrogen will also be analyzed, particularly due to the increased temperatures during drying.

#### 2.2.1. Water Content

**Figure 1:**
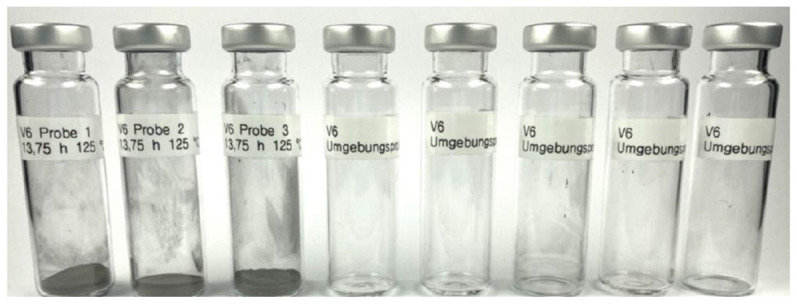
KFT samples with empty reference samples in crimped flasks.

The powder conditioning discussed in this study focused on reducing the water content in order to minimize adsorption effects and moisture bridges, thereby improving the flowability and, ultimately, the processability of the powder. Hence, the gravimetric water content of the analyzed powder will be investigated. Karl Fischer titration (KFT) was used for this, which is one of the methods defined in DIN EN ISO 52928 [35] for determining the moisture content of metallic powders. Three powder samples of 2 ± 0.5 g each were taken. The weight was measured using a PRACTUM 124-1S precision balance. The powder was placed into jars (Figure 1), which were previously conditioned in an inert gas atmosphere. These were then sealed airtight. In addition, five samples were taken using only the ambient atmosphere in the glovebox. The ambient samples were used to calculate the water content in the environment, which was then subtracted from the determined water content of the samples. Five samples were measured, but the water content was only averaged from three; the first two were required for conditioning the titration cell.

The KFT was carried out using the Metrohm 899 Coulometer. The theoretical basis of the method is based on the chemical reaction of the titration solution containing iodine and methanol with sulfur dioxide. Water is consumed during this reaction. The coulometric KFT has the special feature that the iodine is not present in the solution but is generated from an iodine-containing electrolyte during the measurement. First, the sample was placed in a sample holder of the oven. The oven heated the sample to 150 °C to vaporize the water. The measurement started, the sample weight was entered, and the needle was inserted into the sample. The titration solution began to react with the released water. At the same time, iodine was produced, and the amount could be determined via the applied voltage. The measurement was considered complete when the voltage difference between the electrodes decreased, which is indicative of unreacted iodine in the solution, meaning there is no longer enough water for the reaction. The determined water contents can then be noted in gravimetric ppm. Finally, the water content of the three measured samples was averaged. Another value that was noted is the “drift”. This indicates by how many ppm the result is corrected due to escaping gas flow. This must be similar for all measurements, but must always be less than 10 µg/min.

The water content of the undried powder was analyzed before the first and after the last test in order to rule out any possible influence on the results. As not all measurements could be carried out immediately after powder conditioning, but some must be taken on the following day, we checked whether the jars of the KFT measurement were actually airtight or whether the powder became moister again after a longer storage period. For this purpose, one exemplary experiment of the test plan was repeated and twice the number of samples were taken. One of these was measured directly, the other was sealed and stored for seven days under normal room air.

As only three measured values were collected, we decided to display the range instead of the standard deviation. The measurement accuracy of the method is difficult to determine as it depends on many factors. The condition of the titration solution, the accuracy with which the sample weight is determined, and the conditioning times between the individual measurements and the operator are decisive factors. The former cannot be estimated without further actions. The weight was determined with a measurement accuracy of 0.1 mg. Conditioning times of 5 min were observed between each measurement. The measurements were all carried out by one person. According to the instrument manufacturer, a measurement accuracy of ±1.5 ppm can be expected if the aforementioned aspects are observed.

The test plan was carried out over a period of two weeks. Room humidity levels between 52 and 66% and temperatures between 19 and 24 °C were measured. The room humidity of the glovebox was between 12 and 22% and the oxygen content was between 1.5 and 4%. In addition, further test runs were carried out to investigate various aspects in more detail. These aspects are presented as follows:

1. The first test run was carried out to check the necessity of the protective gas atmosphere in relation to the efficiency of the drying process. For this purpose, one exemplary test run was repeated in an ambient atmosphere. The shortest test was selected, as this is probably where the difference, if any, can best be seen.
2. The second run also took place in an ambient atmosphere. This aimed to investigate whether there is increased oxygen uptake when there is more oxygen in the atmosphere. For this purpose, an experiment was carried out with extreme values in order to recognize any increase in oxygen as accurately as possible.
3. As one aim of the work was to formulate guidelines for powder conditioning, a test was also carried out on powder storage itself. For this purpose, the dried powder from the exemplary test was divided into two different powder bottles, one made of metal and one made of plastic, and stored for a week. This allowed for the influence of storage on the water content of the powder to be categorized.

#### 2.2.2. Flowability

**Figure 2:**
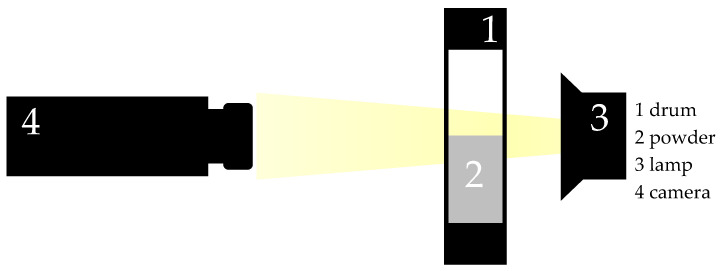
Principle of rotating drum analysis.

One primary focus of the investigation was to improve the flowability of the analyzed fine metal powder. A suitable criterion must therefore be defined. Flowability is not a direct measurement, but rather an indication of how easily a powder can flow freely. Typically, the flow rate through a Hall flow meter according to DIN EN ISO 4490 [36] is used for this purpose. However, this method is unsuitable due to the small particle size distribution and the correspondingly poor flow properties. Therefore, a GranuTools Granudrum using the rotating drum method was used [37], which is better-suited to the MBJ process. The principle of this method is illustrated in Figure 2. A transparent drum (1) was filled halfway with powder (2) and rotated along its axis. This creates an angle of repose for the powder. The drum was backlit to enhance contrast for the camera (3), which captured individual images of the powder during the measurement process (see Figure 2).

**Figure 3:**
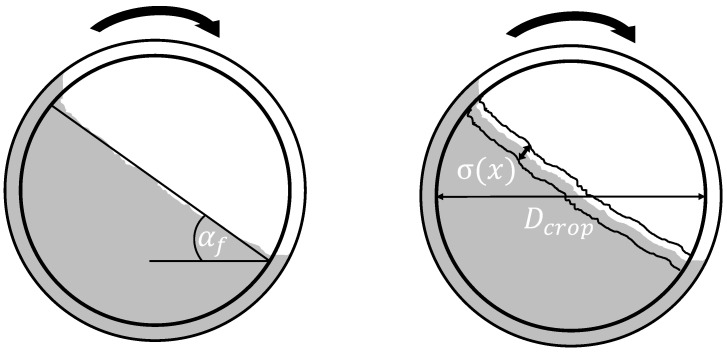
Determination of the dynamic angle of repose and the cohesion index.

One drawback of the method is the challenge of comparability due to the multitude of different measurable parameters [37]. The dynamic angle of repose $\alpha_{f}$ and cohesion index (CI) values were selected for this study (see Figure 3). The dynamic angle of repose describes the flow angle, as depicted in the left-hand side of the illustration at certain rotating speeds.

The cohesion index was determined from the drum diameter used $D_{crop}$ and the fluctuations in the powder surface $\sigma \left(x\right)$ were determined using Formula (1) [37]:

(1) $$CI=\frac{1}{D_{crop}}\int \sigma \left(x\right)dx$$

Both values are strongly dependent on the rotational speed of the drum, which is why they are often specified as a function over a certain range [37]. However, only one sensible speed was selected for the evaluation in the experiments to compare the values more easily. In this case, 6 rpm was selected and converted into a translational speed of 30 mm/s, which corresponds to the coating speed of the MBJ printer used for this work (see Section 2.5).

**Figure 4:**
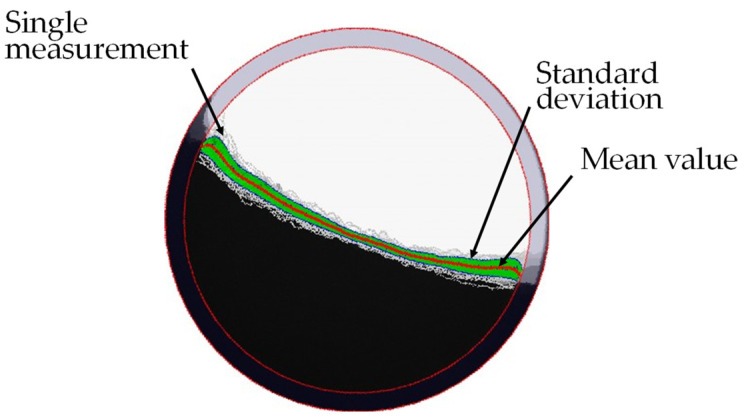
Example of granudrum measurement.

Eighty images were taken for each measurement. The direction of rotation of the drum was changed after 40 images. A total of three powder samples were also measured here, meaning that a total of 240 measured values can be averaged for the dynamic angle of repose α_f_. For the cohesion index CI, only two measured values were collected per measurement, one for each direction of rotation. The result was therefore averaged from six measured values. According to the manufacturer’s experience, the measurement accuracy of the method is 1.67%, which in this case corresponds to a range of 0.52–0.58°. The many measured values for the dynamic angle of repose are necessary here, as the angle was determined using an idealized straight line on the powder surface. This adjustment is difficult with a powder that generally has poor flowability (see Figure 4), as the powder surface has a curved shape.

#### 2.2.3. Impurity Levels

Due to the titanium alloys’ tendency to absorb oxygen and nitrogen and the increased temperatures (up to 200 °C) to which the powder is exposed during conditioning, these two elements were measured using infrared absorption (oxygen) and thermal conductivity cells (nitrogen). Nevertheless, no active oxidation is to be expected at temperatures below 200–300 °C [38].

As with the KFT, the measurement accuracy depends on a number of factors. The main factors are the state of the contactor reagent and the catalysts, the measurement accuracy of the sample weight, the pause times and cleaning between the individual measurements, and the calibration of the measuring device. The elemental measurements were carried out collectively on one day. All reagents were checked optically beforehand. The sample weight was determined with a measurement accuracy of 0.1 mg. The pause times between measurements, which are problematic due to the cooling of the oven, were kept below 10 s. The machine was cleaned after every three samples. Calibration was carried out on the day of measurement using calibration standards. The manufacturer does not specify an exact measurement accuracy, but states that the range of the calibration samples should be taken as a reference value. According to this, the measurement accuracy is 0.006%.

### 2.3. Powder Conditioning

**Figure 5:**
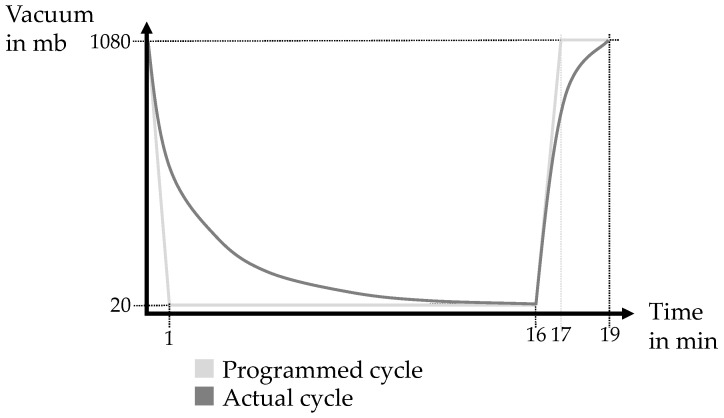
Diagram of the vacuum cycle over time.

A vacuum-drying oven from Memmert VO49 was used for powder conditioning. For this, a fixed vacuum cycle was defined before the experiment was carried out, which was used for all experiments. As illustrated in Figure 5, the maximum vacuum is 20 mb, which can be drawn within 16 min. The pressure was released to ambient pressure after a further three minutes, so that the entire cycle took 19 min. This process was then repeated according to the drying time of the test.

The powder for all experiments came from one container. Before each test, the powder was mixed with a shovel to ensure the same conditions were maintained. Then, 400 ± 3 g of the powder was weighed using a KERN PCB 1000-1 precision balance and placed into a stainless-steel container. This was placed in the drying oven and the program corresponding to the test was started. After the cycle, the oven was first allowed to cool down to room temperature while a vacuum was maintained. The powder container was then returned to the glovebox and mixed again. The sampling using a spoon was carried out according to ISO 8213 [39]. The initial powder moisture content was kept constant by using mixed powder from the same container for all tests. In addition, powder was used that was already stored under normal room air for a longer period of time, which was therefore expected to have reached the maximum moisture content for this powder. The room or powder temperature and room humidity also had an effect. These cannot be changed, but are documented for each test, in accordance with DIN 52928, using a hygrometer. Other disturbance variables, such as measurement errors, were minimized by ensuring that each measurement was carried out at least three times, always by the same person. As factor levels were defined for the statistical test plan, preliminary tests on powder-drying were carried out. Firstly, the powder moisture content of the undried and powder-dried samples, according to a reference at Fraunhofer IAPT (80 °C for 48 h under ambient air), were analyzed using KFT. The values are shown in Table 2. It should be mentioned that the water contents were already comparatively low. When processing coarser powders, this would hardly result in any difference in processing. Due to the comparatively poor flowability of the fine powder fractions, drying is relevant here.

This measurement therefore shows no significant change in the water content under this drying method. Next, preliminary tests were used to narrow down the range for the factor levels. It is assumed that drying in a vacuum oven is significantly more efficient. For this reason, five freely selected preliminary tests were carried out, in which the temperature was varied between 0 and 200 °C and the time between 2 and 15 h. An exemplary result is shown in Table 3. In this case, a more significant difference can be recognized, despite the much shorter time and lower temperature.

With these reference values, the range of the factor time was set to 2.25–13.75 h and the factor temperature to 50–200 °C. Furthermore, it is assumed that the drying relationship is not linear, which is why at least three factor levels were analyzed.

### 2.4. Statistical Design of Experiments (DoE)

The influencing, setting, and disturbance variables for the test plan are shown in Table 4.

Temperature and time were used as setting variables for the test plan. The atmosphere in which drying takes place was kept constant in order to reduce the scope of the test plan. Only an inert gas atmosphere was used due to the safety aspect of handling titanium powder. Nevertheless, a comparison test was carried out with a normal ambient air atmosphere in order to compare the drying quality.

A full-factorial, partial-factorial, or central-composite design (CCD) test plan would be suitable to characterize the given system. The former was not feasible due to the number of experiments. CCD designs always consist of a (partial) factorial, a center point (CP) and star points. With CCD plans, a distinction is usually made between central-composite-face-centered (CCF) and central-composite-circumscribed (CCC) plans [40].

**Figure 6:**
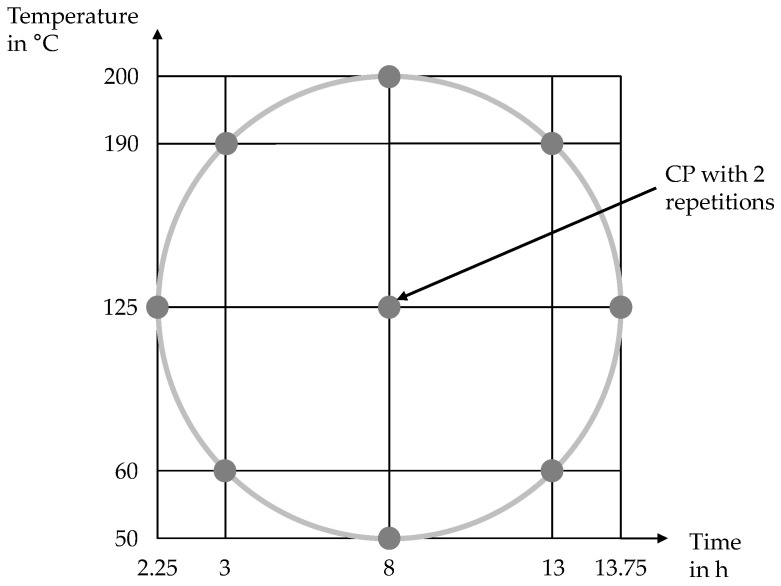
Graphical representation of the experimental design.

By using the CCC plan, the influencing variables of an original 2^2^ test plan could be analyzed at 5 factor levels, for example. As the type of correlation between the variables is not yet known, any quadratic correlations can be easily mapped. If the star points are in the center, between the respective factor levels, this is also referred to as an orthogonal plan, as the regression coefficients are calculated independently of each other. This means that there is no mixing of the interactions [40]. Figure 6 shows the experimental design. A total of eleven experiments were carried out, three of which reflect the CP.

In order to minimize systematic errors, for example due to trends, the tests are randomized, i.e., carried out in a random order. The corresponding test plan is shown in Table 5 and used to examine the following hypotheses:

1. Based on the literature and underlying physical effects (e.g., moisture bridges), it is assumed that the longer and warmer the drying process, the better the flowability and, correspondingly, the lower the cohesion index and water content.
2. The water content and the drying time show an exponential relationship.
3. According to the literature, the oxygen and nitrogen content in the powder does not significantly increase at temperatures under 200 °C.

The Modde^®^ software from Sartorius AG (v13.0.0.24874) was used to analyze the test plan. In the first step, all measurement results were imported. The model, in this case, multiple linear regression, was then selected. The summary of fit (SoF) plot, the analysis of variance (ANOVA), and the lack of fit (LoF) plot were analyzed and evaluated for all target variables.

### 2.5. Binder Jetting System

In order to validate the optimized powder conditioning in practice, test components were produced with unconditioned powder and powder that was dried using the method with the lowest water content. MBJ was carried out on a Digital Metal (now Markforged, Inc., Watertown, NY, USA) DM P2500 system using the C20 binder. The coating was applied via a blade system under vibration. The printing parameters are listed in Table 6:

## 3. Results and Discussion

This section describes the procedure for analyzing the test plan. The optimization of the model is also discussed in more detail. The measured values are rounded to two significant figures in accordance with DIN 1333. The aim of the study is to quantify the water content and associated variables in the powder-drying process, as well as to find an optimized conditioning strategy based on vacuum-drying. In addition, an MBJ print job with undried powder and one with optimized dried powder were compared to validate the conditioning strategy.

### 3.1. Statistical Evaluation

**Figure 7:**
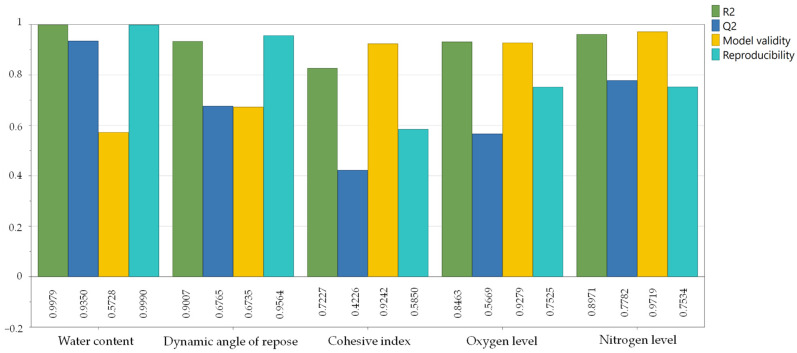
Optimized SoF for all target variables.

Firstly, all measured values were checked for significance in order to subsequently develop models where possible. The initial observation of the SoF plot, ANOVA and the LoF plot showed that an initially significant model could be created (see Appendix C). However, both the SoF and the LoF need to be optimized. This can mainly be achieved by removing outliers, transforming the values and sorting out irrelevant model terms. Firstly, the recorded measurement data were analyzed in the replicate plot, and the range of the CP, which should be as small as possible, was compared with the range of the remaining measured values. This was the case for the water content and the dynamic angle of repose, but the scatter of the measured values of the other variables was significantly higher. The range must therefore be included in the evaluation of the effects. Outliers were checked using the different residual plots. Here one or two experiments per variable were removed. In addition, the observed vs. predicted plot was used to determine the deviation of individual trials from the created model. In order to assess whether a transformation of the measured values is necessary, the histograms were analyzed and a skewness test was performed. The measured values are all normally distributed, so they were not transformed. The next step involves removing non-significant influencing variables or interactions, and therefore also the corresponding model terms. The effect diagrams and coefficient plots were analyzed for this purpose. The optimized SoF is shown in Figure 7.

In order to include the range, the same experimental design was edited again, but this time the measurement repetitions were entered as experimental repetitions (see Figure A8. This shifts the degrees of freedom, but the significance of the measured values can be better assessed. A re-analysis of the LoF shows a significantly higher pure error, i.e., the error in the repetitions, for the target variables’ cohesion index, oxygen content, and nitrogen content. After the optimization, it can be stated that no significant model can be generated for these measured values in the sense of the SoF, as neither influencing variable appears to have a significant effect on the target factors. However, significant models could be created for the water content and the dynamic angle of repose. These are explained in more detail in the following subsections.

### 3.2. Water Content

Table 7 shows the values determined for the gravimetric water content (w), as well as the range and the relative range. At first glance, the relative range of the measurement results appears to be comparatively high and is considered when analyzing the effects. The repeatability of the CP, on the other hand, is significantly smaller than the range.

The water content of the undried powder that was also measured at the beginning of the test series, and was 51.4 ppm. At the end of the test series, its water content was 51.1 ppm, showing no significant difference. The water content of test 6 was 14.5 ppm. After one week of storage in the glass sample bottles, the water content was 15.8 ppm. This change is significantly smaller than the range of the individual measured values. Nevertheless, we noted if a sample was not measured until the following day.

A significant model could be created for the water content with the help of the analysis in Modde^®^. By including the range in the model, it was also possible to create a model that is significant as defined by the SoF. The equation of the multiple linear regression for the water content is shown below (Formula (2)). This consists of the determined coefficient and the factors and interactions labelled as significant.

(2) $$Y=61.1427-0.11267\cdot Temp-5.6265\cdot t-0.000006\cdot {Temp}^{2}+0.1491\cdot t^{2}+0.0098\cdot t\cdot Temp$$

**Figure 8:**
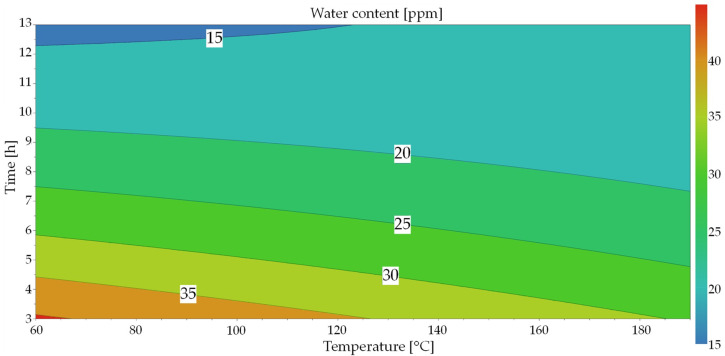
Response contour plot for water content.

This equation was used to create a contour plot (Figure 8) based on the model prediction. This shows the water content as a function of both factors. This can be used to estimate the effect or the effect size of the two factors. Only the area covered by the tests is shown and the intermediate values are interpolated. An extrapolation of the values is associated with uncertainties due to the physical conditions [41]. At first glance, the result of the prediction looks plausible. The higher the temperature and the longer the time, the lower the water content of the powder. It can also be seen that time has a greater influence than temperature, which can also be recognized by the size of the coefficients. The non-linear relationship between the variables, which can be seen from the curved contour lines, is also clearly recognizable.

A significant model for the prediction of the water content of Ti-6Al-4V powder could be created for powder-drying. An additional plot in Appendix A show the general correlation between drying time and water content.

### 3.3. Flowability

When observing the measurement, it is noticeable that the range is so high, because powder clogs on the glass of the camera system as the powder surface or a powder agglomeration piles up, which then breaks out into pieces.

**Figure 9:**
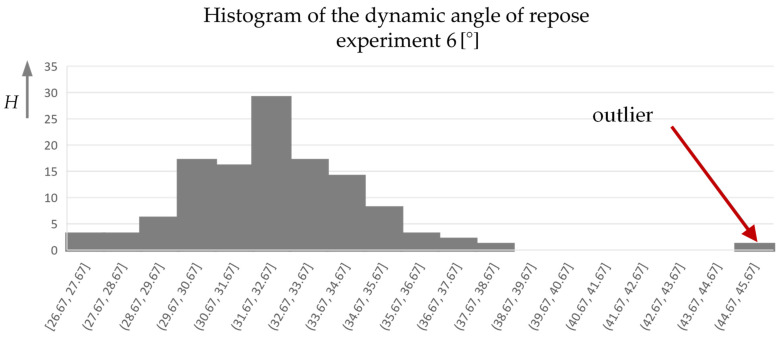
Histogram of the dynamic angle of repose of experiment no. 6.

For this reason, histograms of the dynamic angle of repose over the absolute frequency (H) were created, which were used to remove isolated outliers (see Figure 9).

The measurement data are shown in Table 8. A first look at the measured values reveals that the differences between the individual measured values appear to be smaller than the average range. The range must therefore also be included in the evaluation of the test plan at this point.

An initially significant model could be generated for the dynamic angle of rest by considering the span. The model equation is shown in Formula (3):

(3) Y=37.0691−0.0178·Temp−0.43337·t+0.0014·t·Temp

**Figure 10:**
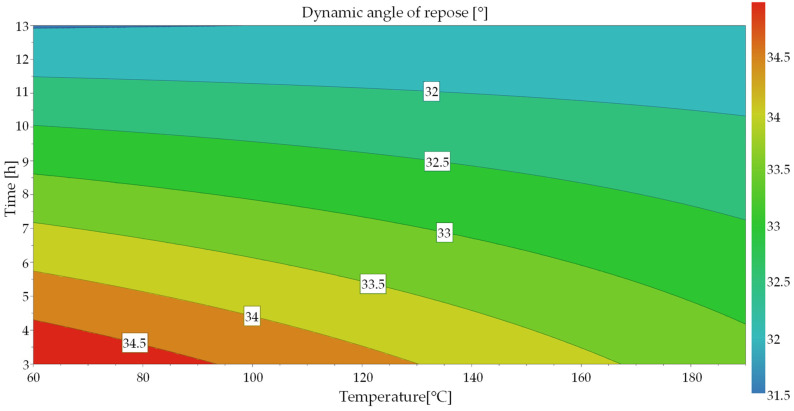
Response surface plot for dynamic angle of repose.

The response surface plot shows that the dynamic angle of repose is almost linearly dependent on time and temperature (see Figure 10). Similar to the water content, time has a stronger influence.

No significant model could be created for the cohesion index (CI). However, as this is directly correlated with the water content and the resulting moisture bridges, as shown in Section 1, it can be assumed that the reproducibility of the results is not sufficient to map the difference. An additional plot in Appendix A show the general correlation between flowability and water content.

### 3.4. Impurity Levels

The measured oxygen (O) and nitrogen (N) content is shown in Table 9.

With the methods used, no temperature- or time-dependent influence on the chemical components can be determined. This is consistent with the findings from Section 2.2.3. showing that titanium shows no active oxidation below 300 °C. Since old powder was used, it can be assumed that the passive oxidation layer had already fully formed in this powder.

### 3.5. Validation

**Figure 11:**
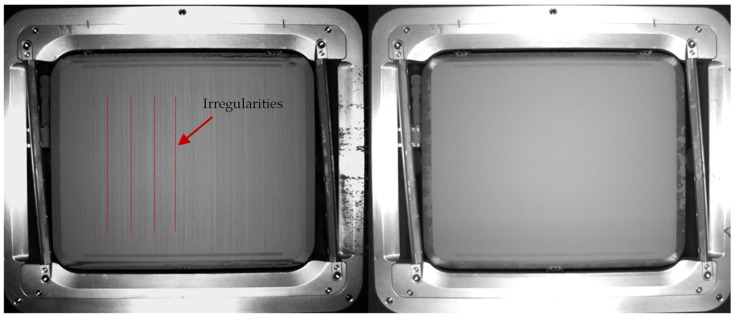
Comparison of the undried (**left**) and conditioned (**right**) powder beds.

**Figure A1:**
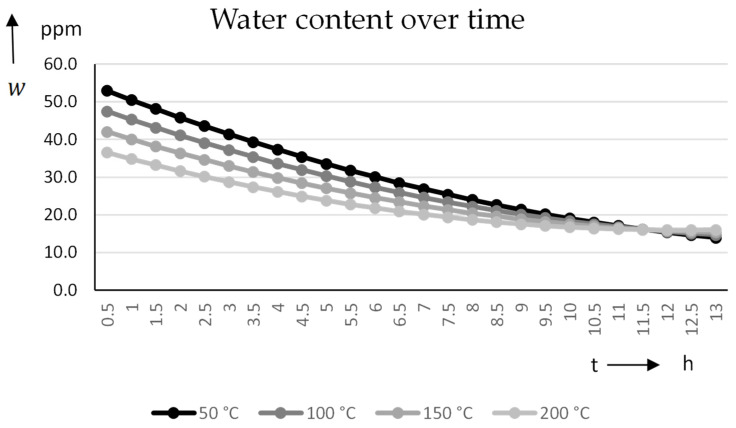
Water content over time.

**Figure A8:**
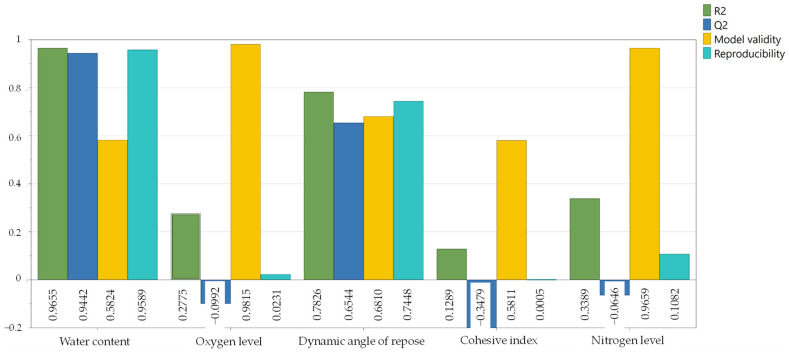
SoF plot of all target values with the included range.

Based on the models that were created, an optimized drying cycle was selected, which was then validated against unconditioned powder. According to the results, drying at 200 °C for 13 h would be the best way to generate the lowest possible water content and high flowability. As the powder is present as bound capital during drying, especially in an industrial process chain, the throughput of the oven must also be considered. For this reason, the course of the water content at 200 °C is considered (Figure A1). After a 6 h drying time, a value of 21.8 ppm was predicted. After 13 h, the value dropped further, to 16 ppm. As the difference in water content decreases sharply after 6 h, this time period was selected. Firstly, the powder beds were visually compared (see Figure 11).

It is noticeable that the powder bed with the new conditioning already appears much more even and uniform, while the reference powder bed looks very streaky.

**Figure 12:**
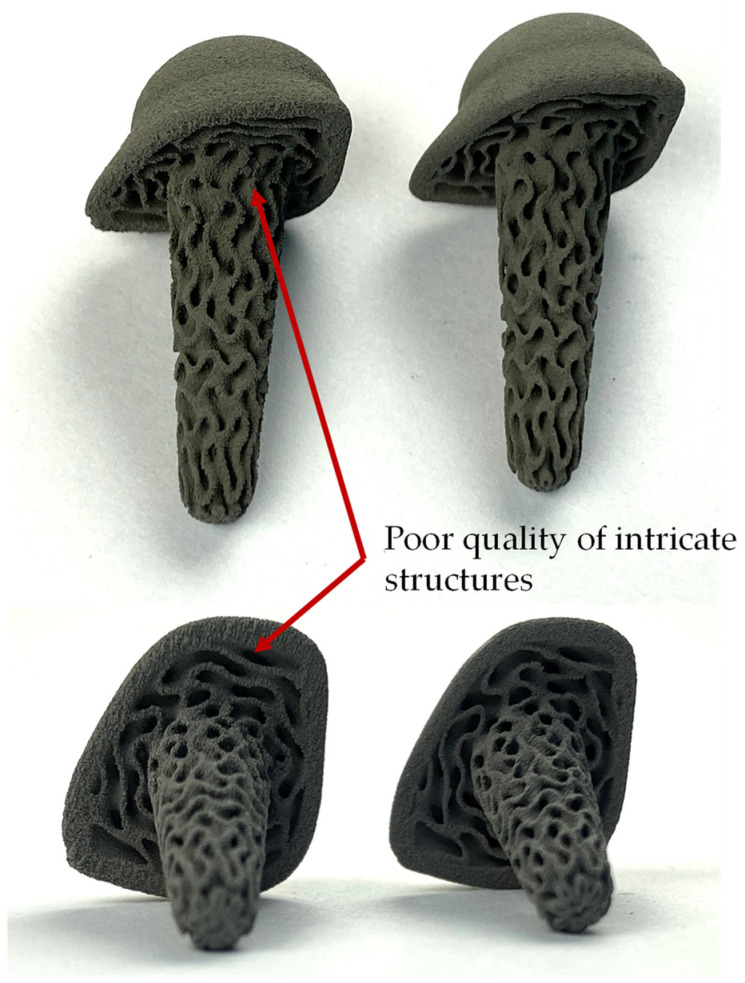
Comparison of demonstrator parts with undried (**left**) and optimized (**right**) powder conditioning.

Demonstrator components were also produced to provide a visual impression of the green part’s quality (see Figure 12). Here, too, the components look more uniform with the new powder conditioning. Especially on the edges and underside of the rod end, the green part with undried powder has a rough structure. This can also be observed at the edges of the structure. In some cases, small pieces of the structure are missing. The green part made from optimized conditioned powder, on the other hand, is of significantly better quality and the complex structure could be reproduced much more accurately.

**Figure 13:**
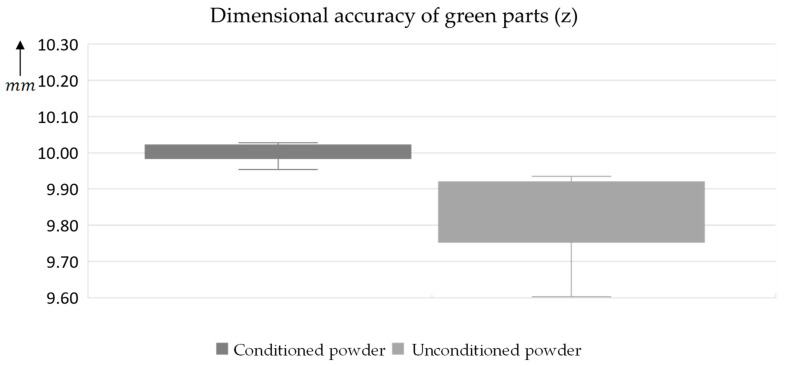
Dimensional accuracy of the test cubes with undried powder and optimized conditioning.

**Figure 14:**
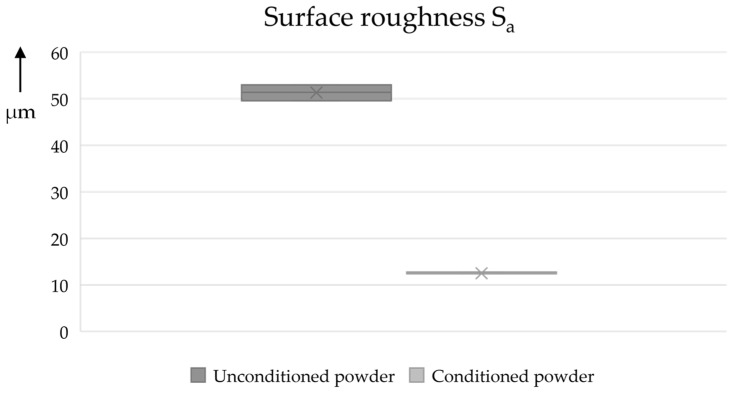
Surface roughness Sa for samples fabricated from unconditioned and conditioned powder.

Furthermore, 10 mm test cubes were produced and analyzed to study their dimensional accuracy (see Figure 13). It can be seen that the components produced with the conditioned powder have a significantly smaller range (±1.5 vs. 0.3%). Similar differences can also be observed in the x and y directions of the test cubes (see Appendix B). In addition, a 3D profilometer (Keyence VR-6000) was used to determine the surface roughness S_a_ in accordance with ISO 25178 [42] (see Figure 14). Here, too, the conditioned powder shows a lower average height (from 12.4 to 53 µm) and a lower relative range of values (from 2.8 to 6.6%).

## 4. Conclusions

The aim of the present work was to compare different drying strategies, to investigate correlations, particularly those regarding the flowability of the investigated powder, and to find an optimized conditioning strategy. The following conclusions can be drawn from the analyses of the hypotheses from Section 2.4:

1. The hypothesis that a longer and warmer drying period leads to an improvement in flowability and a reduction in water content can be accepted. An improvement in the cohesion index cannot be proven with the present method. After analyzing the drying model, an optimized drying cycle of 6 h at 200 °C and a defined vacuum cycle was determined.
2. As assumed, the water content and the drying time show an exponential relationship, as is usual for equalization processes.
3. The oxygen and nitrogen content have not changed significantly.

It can be stated that the process of conditioning Ti-6Al-4V powder notably improves its flowability. Before initial use, it is advisable to dry new powder. Given the uncertain delivery and storage times, the moisture levels in new powder can vary significantly. Significant enhancements in the quality of the green parts, particularly in terms of surface texture and dimensional precision, result from drying the powder. The conditioning also aids in reducing printing errors. It is worth noting that the conditioning duration has a more pronounced effect than the temperature. The authors assume that with each repetition of the cycle, as shown in Figure 5, a “portion” of water is removed from the powder. More time therefore means more “portions” are removed, while higher temperatures lead to larger quantities of vaporized water per cycle. Based on the measured results, it can be assumed that the number of cycles (ergo the number of “portions” removed) has a greater influence than the temperature amplitude (quantity of the “portions”). Drying under a nitrogen environment does not prove to be more efficient than drying in ambient atmosphere within the investigated range of moisture content. However, considering safety aspects is still paramount. During the storage of conditioned powder, a notable increase in water content occurs.

As part of the evaluation, a model for the water content of powder, measured with KFT, was created as a function of time and temperature, which showed an expected exponential dependence. It was also possible to demonstrate a significant improvement in the dynamic angle of repose by means of a dynamic drum measurement. Due to the oxygen affinity of titanium, a chemical analysis of the elements nitrogen and oxygen was also carried out using non-dispersive infrared dispersion and thermal conductivity cell measurement. There was no increase in the oxygen or nitrogen concentration. The green part quality of undried and dried powder was also compared. The drying cycle developed here significantly improved the dimensional accuracy of the green parts. A validation of the principle through the production of demonstrator parts shows promising initial results.

Subjects of future work will include investigating how the developed conditioning strategies affect the reuse of the material. It could be shown that, for individual drying cycles, there is no increase in oxygen or nitrogen. Nevertheless, this cannot be ruled out for multiple repetitions. In general, there is a lack of in-depth studies on the recycling of titanium and its alloys for MBJ. With specific guidelines for powder conditioning and the reuse of titanium powders, MBJ can become more material-efficient without having to take risks with regard to the reliability of the components, especially in the case of medical components.

## Figures and Tables

**Table 1:** Properties of the investigated powder.

Alloy	Supplier	D_10_ (µm)	D_50_ (µm)	D_90_ (µm)	Apparent Density (g/cm^3^)	Tap Density (g/cm^3^)
Ti-6Al-4V	Tekna	9.43 ^1^	15.6 ^1^	21.3 ^1^	≥2.1 ^2^	≥2.7 ^2^

^1^ Measured using dynamic image analysis; ^2^ manufacturer information; apparent density according to ASTM B527 [33] and Tap density according to ASTM B417 [34].

**Table 2:** Water content of undried and powder-dried samples at 80 °C for 24 h under ambient air.

	Undried Powder	Powder Dried at 80 °C for 48 h
Water content (ppm)	51.4	46.2
Range (ppm)	7.0	0.2

**Table 3:** Water content of powder dried at 80 °C for 24 h under ambient air and at 60 °C for 3 h under vacuum and nitrogen purging.

	Powder Dried at 80 °C for 48 h (No Vacuum, Ambient Air)	Powder Dried at 60 °C for 3 h (Vacuum, Nitrogen Purging)
Water content (ppm)	46.2	39.0
Range (ppm)	0.2	2.1

**Table 4:** Influencing, setting, and disturbance variables for the test plan.

Influencing	Setting	Disturbance
Atmosphere	Time	Ambient humidity
Vacuum cycle	Temperature	Ambient temperature
		Powder moisture content
		Powder temperature
		Errors in measurement

**Table 5:** Test plan.

No.	Order	Time (h)	Temperature (°C)
1	1	3	60
2	8	13	60
3	2	3	190
4	7	13	190
5	6	2.25	125
6	5	13.75	125
7	11	8	50
8	10	8	200
9 (CP)	3	8	125
10 (CP)	4	8	125
11 (CP)	9	8	125

**Table 6:** Printing parameters.

Parameter	Value
Binder	Digital Metal C20 Ink
Layer thickness	42 µm
Print box temperature	80 °C
Powder magazine temperature	70 °C
Print speed	200 mm/s
Powder application speed	30 mm/s

**Table 7:** Measured water contents.

No.	w (ppm)	Range of w (ppm)	Relative Range of w (%)
1	31.4	3.2	10.2
2	39.0	2.1	5.4
3	13.9	3.2	23.0
4	19.1	2.6	13.6
5	25.7	2.8	10.9
6	14.5	2.3	15.9
7	29.6	2.5	8.4
8	12.3	3.8	30.8
9 (CP)	22.1	4.5	20.4
10 (CP)	22.0	5.6	25.4
11 (CP)	21.6	1.7	7.9

**Table 8:** Measured angle of repose and cohesion indices from the experimental design.

No.	αf (°)	Range of αf (°)	Relative Range of αf (%)	CI	Range of CI	Relative Range of CI (%)
1	33.0	2.4	7.3	13.2	0.9	6.8
2	33.1	2.6	7.7	13.5	1.0	7.7
3	31.8	2.3	7.3	12.4	0.3	2.6
4	31.8	2.5	7.7	12.6	0.7	5.9
5	33.2	2.4	7.4	13.2	0.7	5.6
6	32.4	2.1	6.5	12.0	0.9	7.8
7	34.6	2.7	7.7	14.7	1.8	12.0
8	31.1	2.5	8.1	12.3	0.8	6.7
9 (CP)	32.6	2.5	7.7	13.0	0.9	6.6
10 (CP)	32.4	2.2	6.9	12.7	0.7	5.8
11 (CP)	32.8	1.9	5.9	11.9	0.9	7.9

**Table 9:** Measured oxygen and nitrogen contents.

No.	O (wt.%)	Range of O (wt.%)	Relative Range of O (%)	N (wt.%)	Range of N (wt.%)	Relative Range of N (%)
1	0.233	0.007	2.969	0.035	0.001	3.124
2	0.246	0.020	8.251	0.037	0.002	6.605
3	0.231	0.032	13.998	0.035	0.006	16.009
4	0.230	0.019	8.045	0.036	0.003	8.974
5	0.241	0.005	2.188	0.037	0.001	2.804
6	0.243	0.024	10.066	0.037	0.004	9.875
7	0.238	0.031	12.963	0.036	0.005	13.312
8	0.222	0.020	9.060	0.033	0.003	9.144
9 (CP)	0.229	0.029	12.850	0.034	0.005	14.148
10 (CP)	0.238	0.021	8.705	0.036	0.003	9.628
11 (CP)	0.233	0.043	18.336	0.035	0.005	15.475

**Figure A2:**
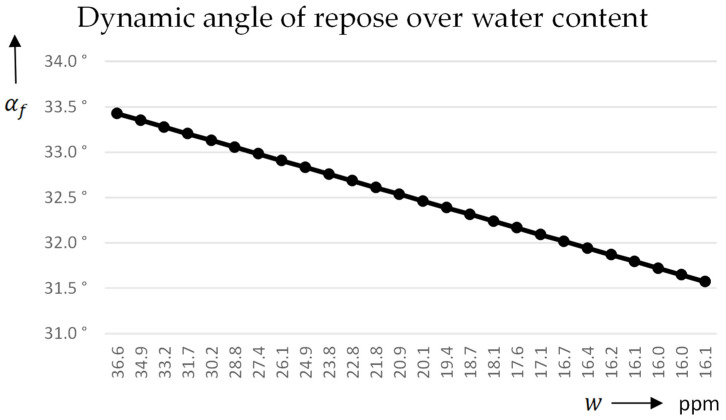
Dynamic angle of repose over water content.

**Figure A3:**
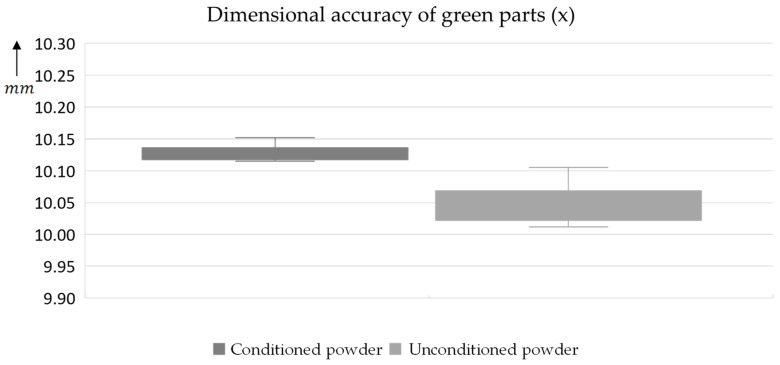
Dimensional accuracy of the test cubes with undried powder and optimized conditioning in the x-direction.

**Figure A4:**
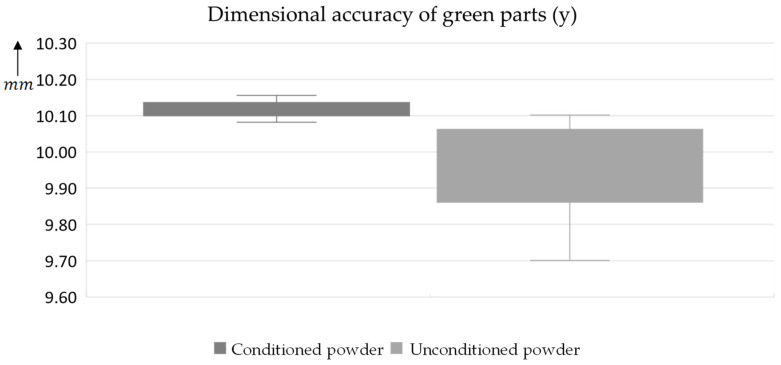
Dimensional accuracy of the test cubes with undried powder and optimized conditioning in the y-direction.

**Figure A5:**
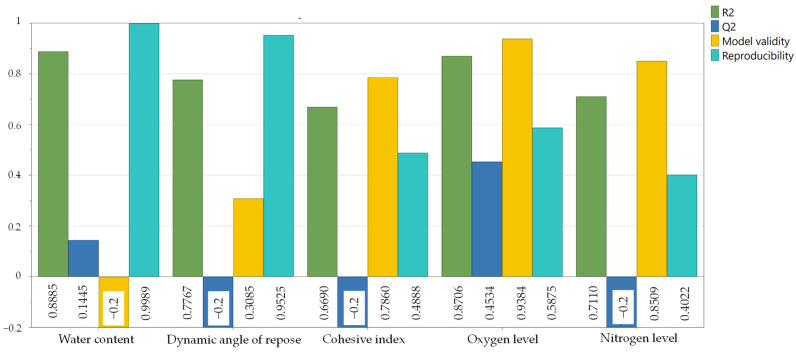
SoF plot for all target values before optimization.

**Figure A6:**
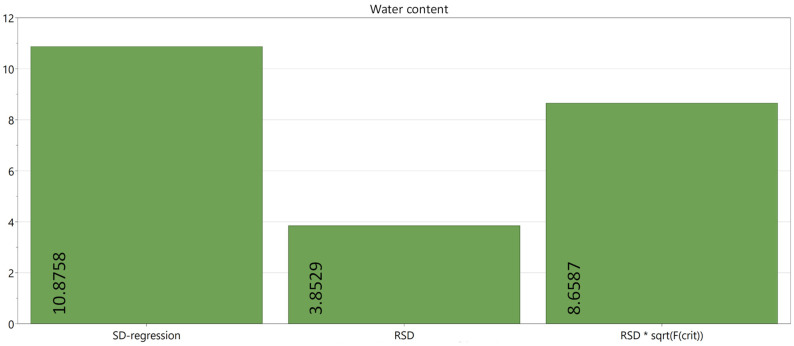
ANOVA plot for water content.

**Figure A7:**
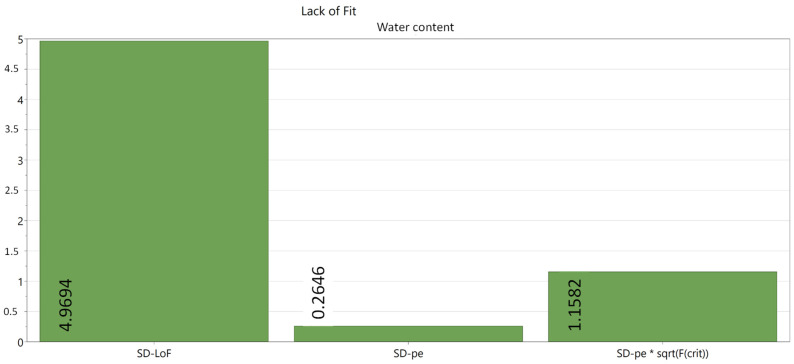
LoF plot for water content.

## Data Availability

Data are contained within the article.

## Appendix A

These additional plots show the general correlation between drying time and water content, as well as flowability and water content.

## Appendix B

These additional plots show the dimensional accuracy of green parts printed with conditioned and unconditioned powder for the x- and y-direction.

## Appendix C

These additional plots show the SoF before optimization, as well as the ANOVA and LoF for the water content. In addition, the SoF plot of all target values is shown, with the range included.
